# The prognostic implications of Notch1, Hes1, Ascl1, and DLL3 protein expression in SCLC patients receiving platinum-based chemotherapy

**DOI:** 10.1371/journal.pone.0240973

**Published:** 2020-10-26

**Authors:** Salomon Tendler, Lena Kanter, Rolf Lewensohn, Cristian Ortiz-Villalón, Kristina Viktorsson, Luigi De Petris

**Affiliations:** 1 Department of Oncology-Pathology, Karolinska Institutet, Stockholm, Sweden; 2 Theme Cancer, Patient Area Head and Neck, Lung, and Skin Cancer, Karolinska University Hospital, Stockholm, Sweden; 3 Pathology Unit, Karolinska University Hospital, Stockholm, Sweden; Tulane University School of Medicine, UNITED STATES

## Abstract

**Objectives:**

The aim was to analyse the tumor expression of Notch1, Hes1, Ascl1, and DLL3 in Small-Cell Lung Cancer (SCLC) and each such biomarker’s potential association with clinical characteristics and prognosis after platinum-doublet chemotherapy (PDCT).

**Material and methods:**

The protein expression of the biomarkers was evaluated using immunohistochemistry. Patients were categorized according to their sensitivity to first line PDCT: with a Progression-free survival (PFS) ≥ 3 months after completion of treatment considered “sensitive” and < 3 months after completion of treatment considered “refractory”. PFS and overall survival were computed using Kaplan-Meier curves with 95% confidence interval.

**Results and conclusion:**

The study included 46 patients, with 21 and 25 of the patients having “sensitive” and “refractory” disease, respectively. The majority of patients had a high DLL3 expression (n = 38), while a minority had Notch 1-high expression (n = 10). The chi-square test showed that there was a statistically significant negative association between Notch1 and Ascl1 expression (p = 0.013). The overall survival for patients with Notch1- high vs. low expression was 8.1 vs. 12.4 months, respectively (p = 0.036). Notch1 expression was an independent prognostic factor in the multivariate analysis (p = 0.02). No other biomarker showed any prognostic impact in this highly selected SCLC cohort. DLL3 is highly expressed in the majority of advanced staged SCLC cases, as expected. In the same patient population, Notch1 expression might have a potential prognostic implication, by driving a non-neuroendocrine differentiation process. Given the small number of cases with Notch1 high expression, the results of this study needs to be confirmed on a larger cohort.

## Background

Small-Cell Lung Cancer (SCLC) is sensitive to first line platinum-doublet chemotherapy (PDCT), with objective response in more than 50% of the patients [1]. Sensitivity to PDCT is usually transient and followed by a PDCT refractory disease resulting in a median progression-free survival (PFS) of around five months [2].

SCLC is characterized by neuroendocrine differentiation and high proliferation [3]. Recent data is also beginning to identify other subtypes of SCLC, one of which includes non-neuroendocrine features [1, 4]. There are several reasons to explore a possible relationship between components of the neuroendocrine molecular make up and prognostic outcomes, especially with respect to how such relationship intersects with the standard of care. A hypothesis is that the Notch signaling pathway is involved in regulating SCLC cells via neuroendocrine differentiation and epithelial to mesenchymal transition [5]. In *vitro*/in *vivo* studies have suggested that Notch1 expression can result in inhibition of cell growth and metastasis of SCLC, however, its role remains unclear [6, 7]. One gene that is regulated by the Notch1 protein is hairy/enhancer of split (Hes1), a basic helix-loop-helix transcription factor, which has been described to prevent transcription of genes that regulate neuroendocrine differentiation [8–10].

The Delta-like protein 3 (DLL3) is a member of the Notch receptor ligand family that is reported to inhibit the Notch receptor activation, and thereby promote neuroendocrine differentiation [11, 12]. In normal cells, DLL3 is only found in the Golgi apparatus and cytoplasmic vesicles [13]. In contrast, in SCLC cells, DLL3 is usually confined to the plasma membrane [13]. DLL3 does not activate signaling in adjacent cells, but only functions when expressed on the same cell as the Notch receptor, in *cis*. When DLL3 binds to the Notch receptor on the cell membrane, Notch1 is relocated to the Golgi apparatus and becomes inactivated [13].

The achaete-scute homolog 1 (*Ascl1)* gene is a basic-helix-loop-helix transcription factor and drives the expression of many oncogenes such as *Sox2*, *MycL* and *BCL-2* [14]. The *ASCL1* gene controls several crucial cellular mechanisms in SCLC, including cell growth and survival [14]. DLL3 expression appears to be a direct downstream target of Ascl1, which interacts with the *DLL3* gene promoter [15]. In *vitro*, Notch1 expressing cells were shown to grow slower and found to be more resistant to PDCT compared to ASCL1 and DLL3 expressing SCLC cells [16]. There is uncertainty regarding the mechanistic roles of the different biomarkers involved in the Notch signaling pathway in SCLC patients, their relative expression in the same tumor samples, and possible associations with sensitivity to platinum chemotherapy, prognosis, and clinical characteristics. Therefore, this study aimed to investigate the expression of Notch1, Hes1, Ascl1, and DLL3 in SCLC, and explore potential prognostic roles after platinum-doublet chemotherapy (PDCT).

## Material and methods

### Data collection

The patient population consisted of SCLC patients who had completed ≥ 1 cycle of PDCT between February 28^th^, 2008 and September 1^st^, 2015, and all data was fully anonymized before performing the analysis. The study was approved by the regional ethics committee in Stockholm (EPN number 2016/8-31) and Stockholm medical biobank (BBK 1693 FUB 2016087). The study was conducted in accordance with the Declaration of Helsinki. All patients were identified from the Swedish Lung Cancer Registry, and data regarding clinical characteristics, treatment patterns and survival outcomes were manually retrieved from each patient’s medical record along with pathology reports.

### Treatment patterns and definition of resistance

Patients receiving PDCT alone in the first line setting (1^st^ L) were defined as having Extensive Disease (ED) and those receiving concurrent chemo- and radio-therapy (CRT) were defined as having Limited Disease (LD). In order to improve the chances to detect differences in outcomes according to biomarker expression, cases were divided into two distinct groups according to the platinum-sensitivity; with a PFS ≥ 3 months after completion of PDCT considered “sensitive” and < 3 months considered “refractory” [3, 17].

### Co-variates

The baseline clinical characteristics obtained for this study were age, gender, smoking status, lab values (Hb, LD, Na, Albumin), stage at diagnosis, performance status (PS), and presence or absence of brain metastasis at diagnosis.

### Definitions of outcomes

The PFS for 1^st^ L was the interval between the start of PDCT and the earliest date of documented clinical or radiological progression according to standard clinical practice, or death. The overall survival (OS) was defined as the interval between start of 1^st^ L PDCT until death. None of the patients were alive at the end of the study.

### Statistical analysis

The associations between biomarker expression and clinical characteristics were analyzed using chi-square test. The calculations for PFS and OS were estimated using the Kaplan-Meier method, and differences in survival distributions were evaluated using the log-rank test. The level of significance was set at p < 0.05. In addition, the clinical factors that were statistically significant in the uni-variate model were further tested in the multi-variate analysis using hazard ratio (HR) and 95% confidence interval (CI) to assess the potential association between clinical and molecular parameters and survival. The analyses were conducted with SPSS program (SPSS IBM corporation version 26.0.; Cary, NC). A power calculation was not performed since this was a descriptive study which included retrospective data.

### Immunohistochemistry

Tumor biopsies were retrieved from the biobank at Karolinska University Hospital. Protein expression was determined by immunohistochemistry on 4-μm-thick formalin-fixed, paraffin-embedded (FFPE) sections. The antibodies used in this study were Notch1 (Cell Signalling Technology, D1E11, Rabbit monoclonal antibody, dilution 1:200), Hes1 (Abcam, 71559, Rabbit polyclonal antibody, dilution 1:800), Ascl1 (Abcam, 206781, Mouse monoclonal antibody, dilution 1:75), and DLL3 (Ventana, 790–7016, Rabbit monoclonal antibody). The Notch1, Hes1, and Ascl1 antibodies were applied manually while the DLL3 staining was performed using an automated immunostaining instrument (VENTANA DLL3 SP347 Assay, Roche Diagnostics). The FFPE sections were de-paraffinized in xylene and rehydrated in alcohol. Antigen retrieval was performed using either citrate buffer pH 6 or EDTA buffer pH 9, depending on the antibody, at 97°C for 20 minutes. For the quenching process, a 30 minute incubation in 0,5% hydrogen peroxidase was performed at room temperature, followed by adding 1% bovine albumin (BSA) for 30 minutes in order to block unspecific antibody binding. The secondary antibodies used were BA-200 anti-mouse for Ascl1 and BA- 1000 anti-rabbit for Notch1 and Hes1 which both were used for 30 minutes at room temperature at a concentration of 1:200.

The next steps included a 30 minute incubation with avidin- biotin enzyme complex followed by a peroxidase substrate DAB, for three minutes.

The sections were counterstained in Mayer´s hematoxylin for one minute followed by dehydration with graded alcohols, xylene and coverslipped with Mountex [18]. For each case, one hematoxylin-eosin staining was performed and a positive/negative control for each protein was carried out according to the manufacturer’s instruction [14]. The evaluation of immunohistochemical stainings was performed by one pathologist (L.K), who was blinded to the clinical data. The number of positive tumor cells was counted under high magnification (x20 and x40) in three random and non-overlapping fields (100 tumor cells per field with a total of 300 tumor cells per specimen) [19].

The scoring of IHC staining in the cases were made into four categories according to the number of positive tumor cells stained; 0: No positive cells, 1; 1–25% positive cells, 2; 26–50% positive cells, 3; 51–75% cells, 4; more than 76% positive cells. The staining intensity was defined as any positivity in the tumor cells of each specimen [19, 20].

Since only DLL3 has a validated cut-off between low vs high expression, this study aimed to establish a cut-off for the other biomarkers based on sensitivity to PDCT using a dichotomizing score with receiver operating characteristic (ROC) curve analysis **S5 Fig** [21]. The hypothesis was that Notch1 and Hes1 high expression would be more “refractory” to PDCT, while Ascl1 high expression be more “sensitive” to PDCT [16].

## Results

### Baseline characteristics of the patients with regards to each biomarker

The study included 46 SCLC patients. The median age of the population was 68 years (IQR 61–76). The majority of patients were women (57%), and most of them (82%) received CT alone in the 1^st^ L, which was in accordance with the proportion of patients with advanced stage disease at diagnosis. A majority of the patients had good performance status (PS 0–1) (63%). The distribution and associations between baseline characteristics according to evaluated biomarker expression is presented in **Table 1.**

**Table 1:** Patient characteristics and median PFS and OS by Notch1, Hes1, Ascl1 and DLL3 low vs. high expression.

	Notch 1		Hes 1		Ascl l		DLL3	
	Low (n = 36) High (n = 10)		Low (n = 3) High (n = 43)		Low (n = 30) High (n = 16)		Low (n = 8) High (n = 38)	
**Median PFS**	7.8	5.1	8.1	7.1	8.1	7.1	5.6	7.4
**95% CI**	(7.1–8.6)	(3.6–9.6)	(7.0–9.2)	(5.1–9.0)	(7.0–9.2)	(5.1–9.0)	(2.5–8.7)	(6.1–8.7)
**Median OS**	12.4	8.1	9.8	11.5	11.1	11.5	10.6	11.5
**95% CI**	(9.8–15.4)	(5.4–10.8)	(8.0–11.6)	(9.1–13.8)	(8.3–13.9)	(4.0–19.0)	(8.9–12.4)	(9.2–13.7)
**Platinum Sensitivity**								
**Low**	13	8	0	21	11	10	4	17
(n = 21)	(36%)	(80%)	(0%)	(49%)	(37%)	(62%)	(50%)	(45%)
**High**	18	2	3	22	19	6	4	21
(n = 25)	(64%)	(20%)	(100%)	(51%)	(63%)	(38%)	(50%)	(55%)
**Age**							*	*
≥ 70 years	23	6	1	28	16	13	4	25
(n = 29)	(64%)	(60%)	(33%)	(65%)	(53%)	(81%)	(50%)	(67%)
<70 years	13	4	2	15	14	3	4	13
(n = 17)	(36%)	(40%)	(67%)	(35%)	(47%)	(19%)	(50%)	(33%)
**Gender**	*	*						
Men	16	4	1	19	13	7	2	18
(n = 20)	(44%)	(40%)	(33%)	(44%)	(43%)	(44%)	(25%)	(47%)
Women	20	6	2	24	17	9	6	20
(n = 26)	(56%)	(60%)	(67%)	(56%)	(57%)	(56%)	(75%)	(53%)
**PS**								
0	10	3	0	13	8	5	2	11
(n = 13)	(28%)	(30%)	(0%)	(30%)	(27%)	(31%)	(25%)	(29%)
1	14	2	1	15	9	7	3	13
(n = 16)	(39%)	(20%)	(33%)	(35%)	(30%)	(44%)	(37.5%)	(33%)
2	9	5	2	12 (28%)	11	3	3	11
(n = 14)	(25%)	(50%)	(67%)		(37%)	(19%)	(37.5%)	(29%)
3	3	0	0	3	2	1	0	3
(n = 3)	(8%)	(0%)	(0%)	(7%)	(6%)	(6%)	(0%)	(9%)
**Stage**								
LD	14	5	1	18	15	4	2	17
(n = 19)	(30%)	(50%)	(33%)	(39%)	(50%)	(25%)	(25%)	(44%)
ED	22	5	2	25	15	12	6	21
(n = 27)	(70%)	(50%)	(67%)	(61%)	(65%)	(75%)	(75%%)	(56%)
**Brain mets**								
Yes	23	5	2	26	20	8	5	23
(n = 28)	(64%)	(50%)	(67%)	(60%)	(67%)	(50%)	(62.5%)	(35%)
No	13	5	1	17	10	8	3	15
(n = 18)	(36%)	(50%)	(33%)	(40%)	(33%)	(50%)	(37.5%)	(65%)

* Statistically significant association between the biomarker and the clinical characteristic.

Abbrevations; PFS- Progression-free survival, OS- Overal survival, Brain mets- Brain metastasis, LD- Limited Disease, ED- Extensive Disease, PS- Performance status, CI- Confidence Interval

### Expression patterns of each biomarker

In the examined SCLC specimen Notch1 expression was predominantly cytoplasmatic which was in accordance with previous reports [22]. Hes1 and Ascl1 were considered positive if expressed in either the cytoplasm or nucleus, whereas DLL3 showed a distinct membranous pattern in the analyzed SCLC cohort in line with literature [18, 19, 23].

**Fig 1:**
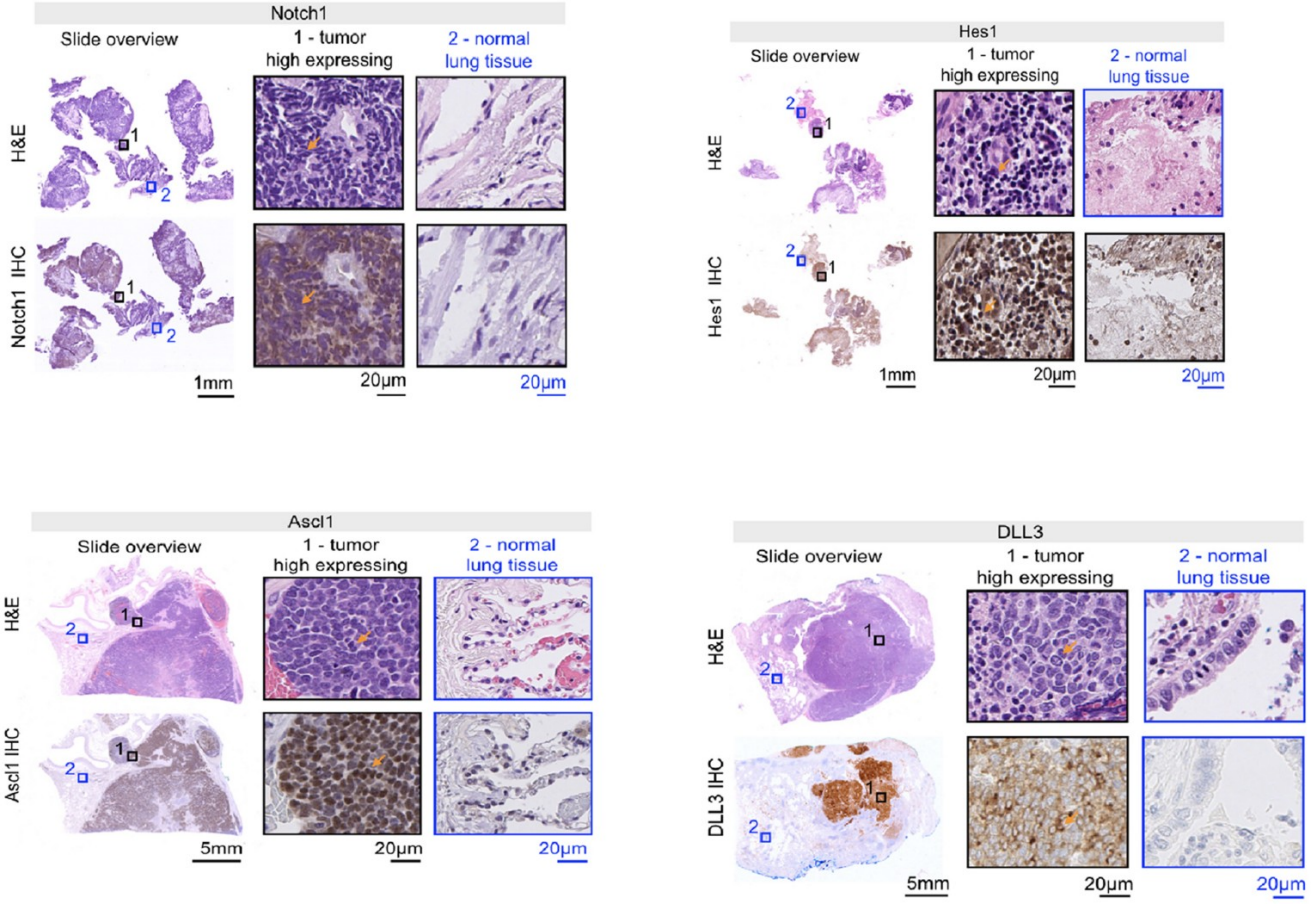
Expression of Notch1, Hes 1, Ascl1 and DLL3. Representative immunohistochemical staining’s. Left panel: overview of the tumor biopsy (SCLC) with Hematoxylin and Eosin (HE) staining (top) and the expression of the different biomarkers (bottom), Middle panel: (1)–The localization of SCLC HE image (top), and the same area for high expression of the biomarker in IHC (bottom). Right panel: (2)–Normal lung tissue within the SCLC HE image (above), and the same are for the biomarker expression (below). (A) Notch1, (B) Hes 1, (C) Ascl1 and (D) DLL3. The arrows point at the representative SCLC cells in the HE images (top) as well as each biomarker (below).

Representative staining’s are presented in **Fig 1A–1D**. The expression pattern for each biomarker is listed in **Table 2.** The positive controls for each biomarker was presented in **S1−S4 Figs.**

**Table 2:** Number of SCLC patients (n = 46) according to proportion of cancer cells positive for Notch1, Hes1, Ascl1 and DLL3 expression.

Number of positive cells	Notch1	Hes1	Ascl1	DLL3
0%	36	3	7	3
(negative)	(80%)	(7%)	(15%)	(7%)
1–25%	4	13	12	6
	(7%)	(28%)	(26%)	(13%)
26–50%	6	7	5	9
	(13%)	(15%)	(11%)	(20%)
51–75%	0	7	7	9
	(0%)	(15%)	(15%)	(20%)
≥76%	0	16	15	19
	(0%)	(35%)	(33%)	(40%)

The cut-off for high vs low biomarker expression was set at ≥ 1% of the neoplastic cells for Notch1 and Hes1, >76% for Ascl1 and > 51% for DLL3.

### Negative association between Notch1 and Ascl1 expression

There was a statistically significant negative association between Notch1 and Ascl1 expression, using the chi-square test. (-0.364, p = 0.013). **S6 Fig** The other biomarkers showed no statistically significant association with each other.

### Subjects with Notch1 low expression had a better prognosis and higher sensitivity to PDCT

**Fig 2:**
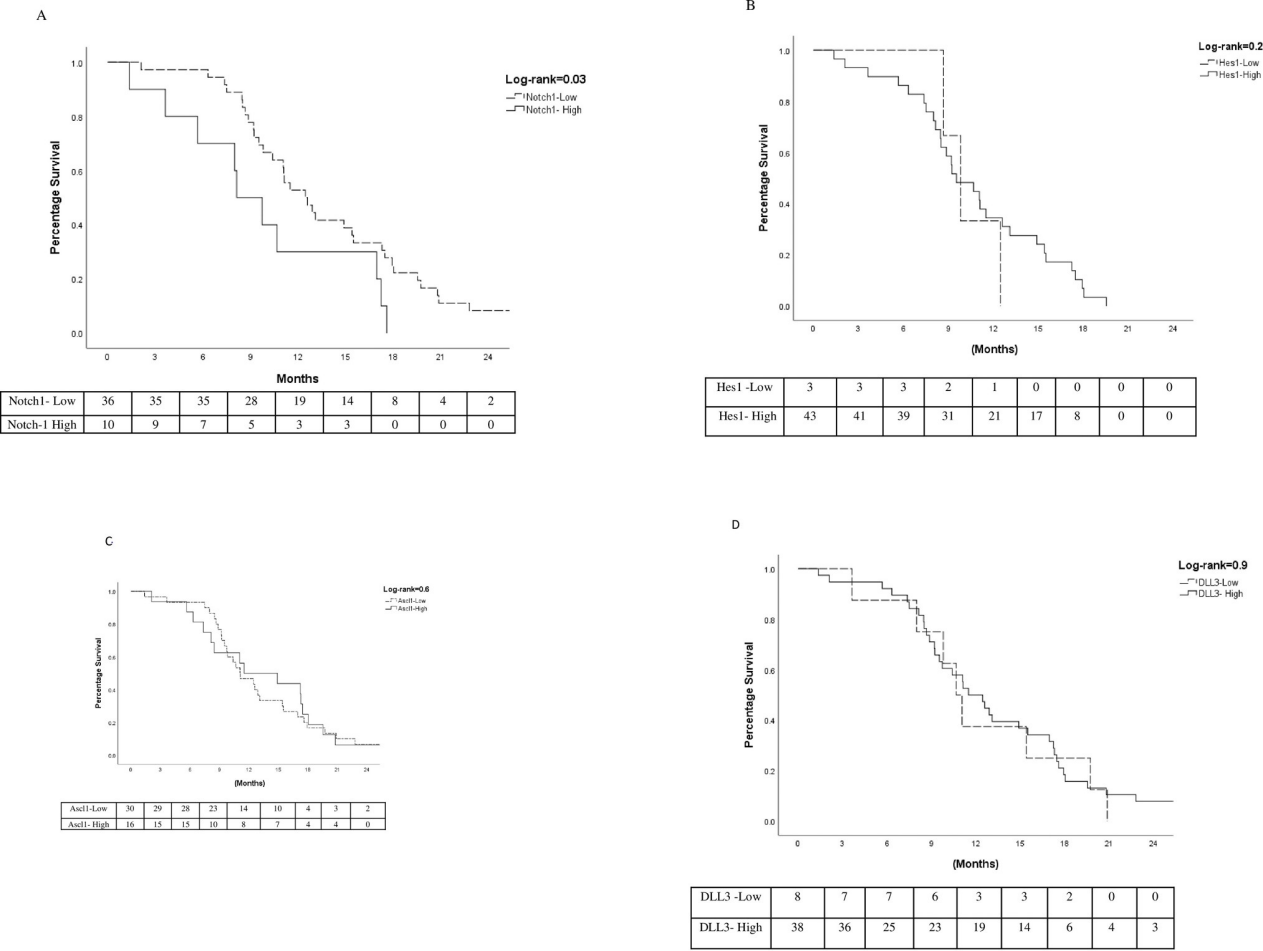
Overall survival Kaplan-Meier curves for low vs high expression of biomarkers. Notch 1 (**A**), Hes1 (**B**), Ascl1 (**C**), DLL3 (**D**).

Patients with a Notch1-low expression had a 4.3 months longer median OS when compared to Notch1- high expression (p = 0.036), and a longer median PFS; 7.8 vs 5.1 months, respectively (p = 0.014). The Hes1, Ascl1 and DLL3 showed no differences in sensitivity to PDCT or prognosis between low vs. high expression. **Fig 2A–2D.** There were also no statistically significant prognostic differences between low vs high expression for Hes1, Ascl1 and DLL3 when stratified for stage of disease.

The clinical characteristics that were statistically significant prognostic factors in the uni-variate analysis were Lactate dehydrogenase (LDH) values and stage of the disease. Notch1 was shown to be an independent prognostic factor even in the multi-variate analysis. **Table 3.**

**Table 3:** Uni- and Multi-variate analysis of Notch1 expression adjusted for stage of disease and Lactate dehydrogenase.

n = 46	Univariate		Multivariate	
	HR (95% CI)	p-value	HR (95% CI)	p-value
**Notch 1**				
**expression**				
High vs Low	2.10 (1.03–4.48)	0.04	2.42 (1.2–5–2)	0.02
**Stage of the disease**				
LD vs ED	0.51 (0.17–0.82)	0.003	0.46 (0.13–0.76)	0.002
**Lab values**				
Ldh (log)	1.12 (1.03–1.25)	0.044	1.14 (1.02–1.49)	0.04

*Abbreviations*: *Lactate dehydrogenase (Ldh)*, LD- Limited Disease, ED- Extensive Disease

## Discussion

The aim of this study was to determine the protein expression levels of Notch1, Hes1, Ascl1, DLL3 in SCLC tumor specimen and the potential association of these biomarkers to platinum chemotherapy sensitivity, prognosis as well as clinical factors in SCLC patients.

This is to the best of our knowledge, the first study to analyze the expression of these four biomarkers in the same patient cohort. This is also the first study, to our knowledge, which validates the use of specific Notch1, Hes1, and Ascl1 antibodies on human SCLC samples, and tries to find cut-offs for low vs high expression based on a biological and clinical rationale.

The cohort was highly selected depending on sufficient biopsy material, received platinum chemotherapy and follow-up data, in order to evaluate the specific aims of this study.

Many of the biopsies were small, which could have limited the interpretation of tumor heterogeneity compared to surgically removed tumors. In addition, the samples size was small (n = 46), and its retrospective design made it difficult to evaluate treatment efficacy by response rates of chemotherapy, according to RECIST criteria.

The proportion of subjects with Notch1 expression was similar to a previous study [23]. In a preclinical triple knockout mouse model, Notch1-low expression was associated with a better prognosis, which is in accordance with our results [16]. However, in another study on surgically resected SCLC samples (n = 125), low Notch1 expression was an unfavorable prognostic factor [22]. That study included only operable early stage SCLC cases, which represent a small subset of patients who are diagnosed with SCLC. The ability to extrapolate these findings to our study is limited since our cohort mainly consisted of cases with advanced stage of disease [22]. In addition, there are no studies that have analyzed Notch1 expression after PDCT, since few SCLC patients are re-biopsied. The concept of re-biopsy in SCLC patients in order to understand the mechanistic changes in protein expression of Notch signaling pathway should be performed on a prospective material.

We found a negative association between Notch1 and Ascl1 expression in our SCLC cohort, which is supported by an earlier study which mechanistically reported that Ascl1 has the ability to reduce Notch1 at both transcription and post-translational level, the later by protein degradation [24].

Our results show that Hes1 expression is found in most of the SCLC cases analyzed. Thus, our results substantiate in *vitro* results from SCLC cell lines where Hes1 was found in cell lines with neuroendocrine features [14]. However, as we could not reveal a significant association between Hes1 and Notch1 expression, it seems that Hes1 is not solely regulated by Notch1, and hence its regulation in SCLC needs to be further investigated [8].

In line with previous results, we found that the majority of cases in our cohort had positive Ascl1 staining and 16% were scored as high expression. There was a positive association between Ascl1 and DLL3, although without reaching statistical significance, as instead reported in a previous study on surgically resected SCLC patients (n = 95) [24]. This discrepancy could possibly be explained by a small number of patients in our study.

The high proportion of patients with positive DLL3 tumor expression on the cell surface, though without prognostic implications, confirms previous results [15, 19]. Hence, DLL3 remains a potential target for future therapeutic agents [15].

## Conclusion

SCLC is a heterogeneous disease with many potential factors that affect sensitivity to platinum-based chemotherapy and prognosis. The small number of Notch1-positive cases precludes any conclusion to be drawn with respect to its prognostic impact. However, patient with a low Notch1 expression in their tumors had a better survival, which makes further studies warranted. The expression patterns of Notch1, Hes1, Ascl1, and DLL3 were similar to previous findings.

## Supporting information

S1 Fig Representative IHC images of positive control for each biomarker. Notch1- Tonsil Cancer.
(TIF)

S2 Fig Representative IHC images of positive control for each biomarker. Hes1- Pancreatic Cancer.
(TIF)

S3 Fig Representative IHC images of positive control for each biomarker. Ascl1- Small Cell Lung Cancer.
(TIF)

S4 Fig Representative IHC images of positive control for each biomarker. DLL3- Small Cell Lung Cancer.
(TIF)

S5 Fig The receiver operating characteristic (ROC) curve analysis for each biomarker Notch 1 (A), Hes1 (B), Ascl1 (C), DLL3 (D), with sensitivity to platinum-doublet chemotherapy as the outcome of interest.
(TIF)

S6 Fig The association between Notch1 and Ascl1 plotted with respect to low vs high expression. (TIF)

## References

[1] Gazdar AF, Bunn PA, Minna JD. Small-cell lung cancer: what we know, what we need to know and the path forward. Nature Reviews Cancer. 2017;17:725 10.1038/nrc.2017.87 29077690

[2] Rossi A. Relapsed small-cell lung cancer: platinum re-challenge or not. J Thorac Dis. 2016;8(9):2360–4. 10.21037/jtd.2016.09.28 27746976 PMC5059314

[3] Garassino MC, Torri V, Michetti G, Lo Dico M, La Verde N, Aglione S, et al Outcomes of small-cell lung cancer patients treated with second-line chemotherapy: a multi-institutional retrospective analysis. Lung cancer (Amsterdam, Netherlands). 2011;72(3):378–83. 10.1016/j.lungcan.2010.09.009 20950888

[4] Rudin CM, Poirier JT, Byers LA, Dive C, Dowlati A, George J, et al Molecular subtypes of small cell lung cancer: a synthesis of human and mouse model data. Nature reviews Cancer. 2019;19(5):289–97. 10.1038/s41568-019-0133-9 30926931 PMC6538259

[5] Morimoto M, Nishinakamura R, Saga Y, Kopan R. Different assemblies of Notch receptors coordinate the distribution of the major bronchial Clara, ciliated and neuroendocrine cells. Development. 2012;139(23):4365–73. 10.1242/dev.083840 23132245 PMC3509731

[6] Wael H, Yoshida R, Kudoh S, Hasegawa K, Niimori-Kita K, Ito T. Notch1 signaling controls cell proliferation, apoptosis and differentiation in lung carcinoma. Lung cancer (Amsterdam, Netherlands). 2014;85(2):131–40. 10.1016/j.lungcan.2014.05.001 24888228

[7] Hassan WA, Yoshida R, Kudoh S, Hasegawa K, Niimori-Kita K, Ito T. Notch1 controls cell invasion and metastasis in small cell lung carcinoma cell lines. Lung cancer (Amsterdam, Netherlands). 2014;86(3):304–10. 10.1016/j.lungcan.2014.10.007 25456735

[8] Nowell CS, Radtke F. Notch as a tumour suppressor. Nature Reviews Cancer. 2017;17(3):145–59. 10.1038/nrc.2016.145 28154375

[9] Ito T, Kudoh S, Ichimura T, Fujino K, Hassan WAMA, Udaka NJHC. Small cell lung cancer, an epithelial to mesenchymal transition (EMT)-like cancer: significance of inactive Notch signaling and expression of achaete-scute complex homologue 1. 2017;30(1):1–10. 10.1007/s13577-016-0149-3 27785690

[10] Marignol LJTCR. Notch signalling: the true driver of small cell lung cancer? 2017 2017:S1191–S6.

[11] Rudin CM, Pietanza MC, Bauer TM, Ready N, Morgensztern D, Glisson BS, et al Rovalpituzumab tesirine, a DLL3-targeted antibody-drug conjugate, in recurrent small-cell lung cancer: a first-in-human, first-in-class, open-label, phase 1 study. The Lancet Oncology. 2017;18(1):42–51. 10.1016/S1470-2045(16)30565-4 27932068 PMC5481162

[12] Owen DH, Giffin MJ, Bailis JM, Smit M-AD, Carbone DP, He K. DLL3: an emerging target in small cell lung cancer. Journal of hematology & oncology. 2019;12(1):61-. 31215500 10.1186/s13045-019-0745-2 PMC6582566

[13] Geffers I, Serth K, Chapman G, Jaekel R, Schuster-Gossler K, Cordes R, et al Divergent functions and distinct localization of the Notch ligands DLL1 and DLL3 in vivo. 2007;178(3):465–76. 10.1083/jcb.200702009 PMC2064846 17664336

[14] Fujino K, Motooka Y, Hassan WA, Ali Abdalla MO, Sato Y, Kudoh S, et al Insulinoma-Associated Protein 1 Is a Crucial Regulator of Neuroendocrine Differentiation in Lung Cancer. The American journal of pathology. 2015;185(12):3164–77. 10.1016/j.ajpath.2015.08.018 26482608

[15] Saunders LR, Bankovich AJ, Anderson WC, Aujay MA, Bheddah S, Black K, et al A DLL3-targeted antibody-drug conjugate eradicates high-grade pulmonary neuroendocrine tumor-initiating cells in vivo. Science translational medicine. 2015;7(302):302ra136-302ra136. 10.1126/scitranslmed.aac9459 PMC4934375 26311731

[16] Leonetti A, Facchinetti F, Minari R, Cortellini A, Rolfo CD, Giovannetti E, et al Notch pathway in small-cell lung cancer: from preclinical evidence to therapeutic challenges. Cellular Oncology. 2019;42(3):261–73. 10.1007/s13402-019-00441-3 PMC12994342 30968324

[17] Ardizzoni A, Tiseo M, Boni L. Validation of standard definition of sensitive versus refractory relapsed small cell lung cancer: a pooled analysis of topotecan second-line trials. Eur J Cancer. 2014;50(13):2211–8. 10.1016/j.ejca.2014.06.002 24981975

[18] Lim JS, Ibaseta A, Fischer MM, Cancilla B, O'Young G, Cristea S, et al Intratumoural heterogeneity generated by Notch signalling promotes small-cell lung cancer. Nature. 2017;545(7654):360–4. 10.1038/nature22323 28489825 PMC5776014

[19] Tanaka K, Isse K, Fujihira T, Takenoyama M, Saunders L, Bheddah S, et al Prevalence of Delta-like protein 3 expression in patients with small cell lung cancer. Lung cancer (Amsterdam, Netherlands). 2018;115:116–20. 10.1016/j.lungcan.2017.11.018 29290251

[20] Fedchenko N, Reifenrath J. Different approaches for interpretation and reporting of immunohistochemistry analysis results in the bone tissue—a review. Diagn Pathol. 2014;9:221-. 10.1186/s13000-014-0221-9 25432701 PMC4260254

[21] Goos JA, Coupe VM, Diosdado B, Delis-Van Diemen PM, Karga C, Belien JA, et al Aurora kinase A (AURKA) expression in colorectal cancer liver metastasis is associated with poor prognosis. British journal of cancer. 2013;109(9):2445–52. 10.1038/bjc.2013.608 24104968 PMC3817339

[22] Kikuchi H, Sakakibara-Konishi J, Furuta M, Yokouchi H, Nishihara H, Yamazaki S, et al Expression of Notch1 and Numb in small cell lung cancer. Oncotarget. 2017;8(6):10348–58. 10.18632/oncotarget.14411 28060745 PMC5354663

[23] Ito T. Intratumoral heterogeneity of Notch1 expression in small cell lung cancer. J Thorac Dis. 2018;10(3):1272–5. 10.21037/jtd.2018.03.61 29707277 PMC5906247

[24] Sriuranpong V, Borges MW, Strock CL, Nakakura EK, Watkins DN, Blaumueller CM, et al Notch Signaling Induces Rapid Degradation of Achaete-Scute Homolog 1. 2002;22(9):3129–39. 10.1128/MCB.22.9.3129-3139.2002 PMC133746 11940670

