# Supplementary figures and images for: The prognostic implications of Notch1, Hes1, Ascl1, and DLL3 protein expression in SCLC patients receiving platinum-based chemotherapy
Source: PLoS One. 2020 Oct 26;15(10):e0240973. doi: 10.1371/journal.pone.0240973 (PMC7590528; doi:10.1371/journal.pone.0240973)

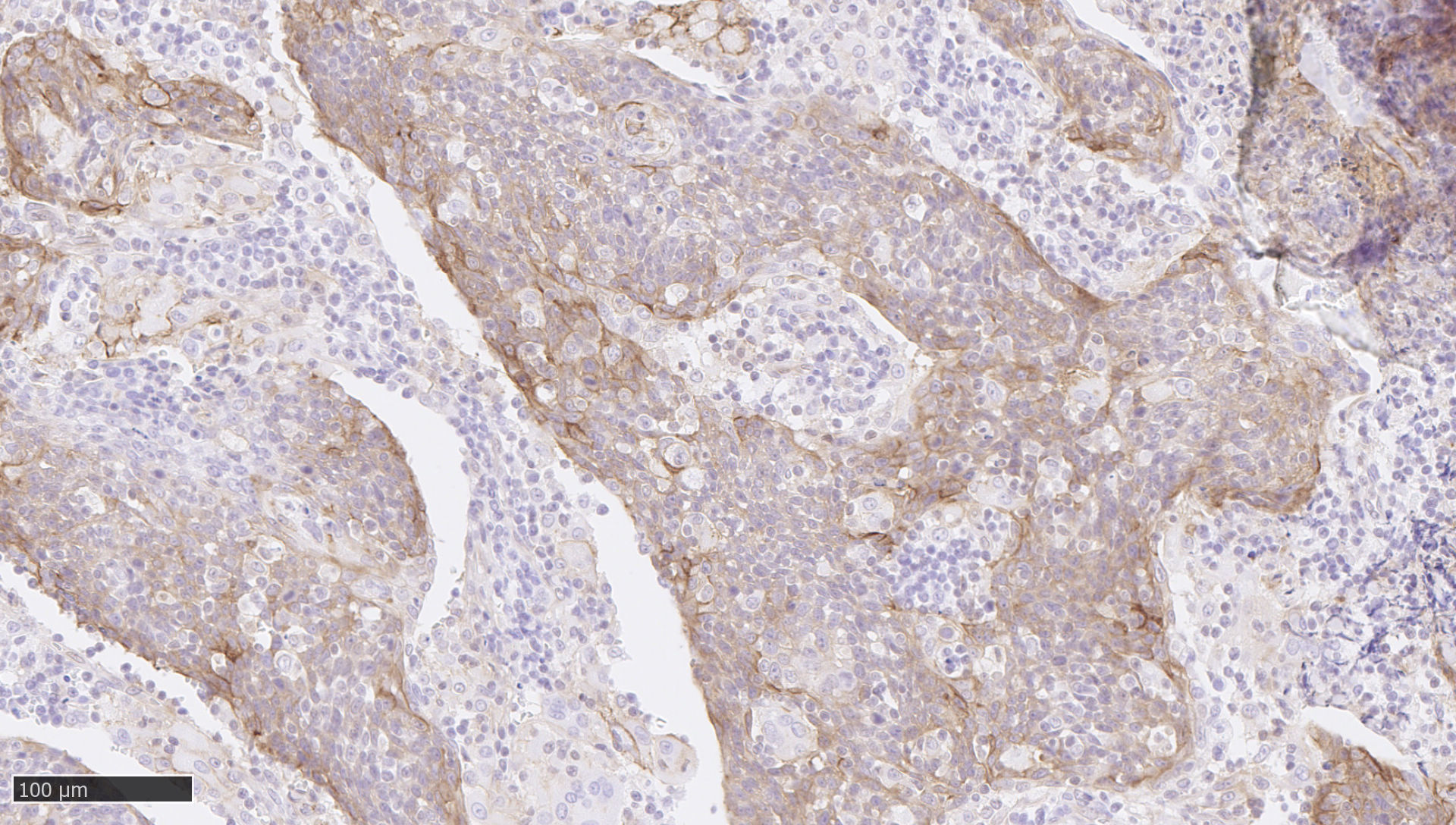

Supplement: S1 Fig — Notch1- Tonsil Cancer. (TIF) [file pone.0240973.s001.tif]

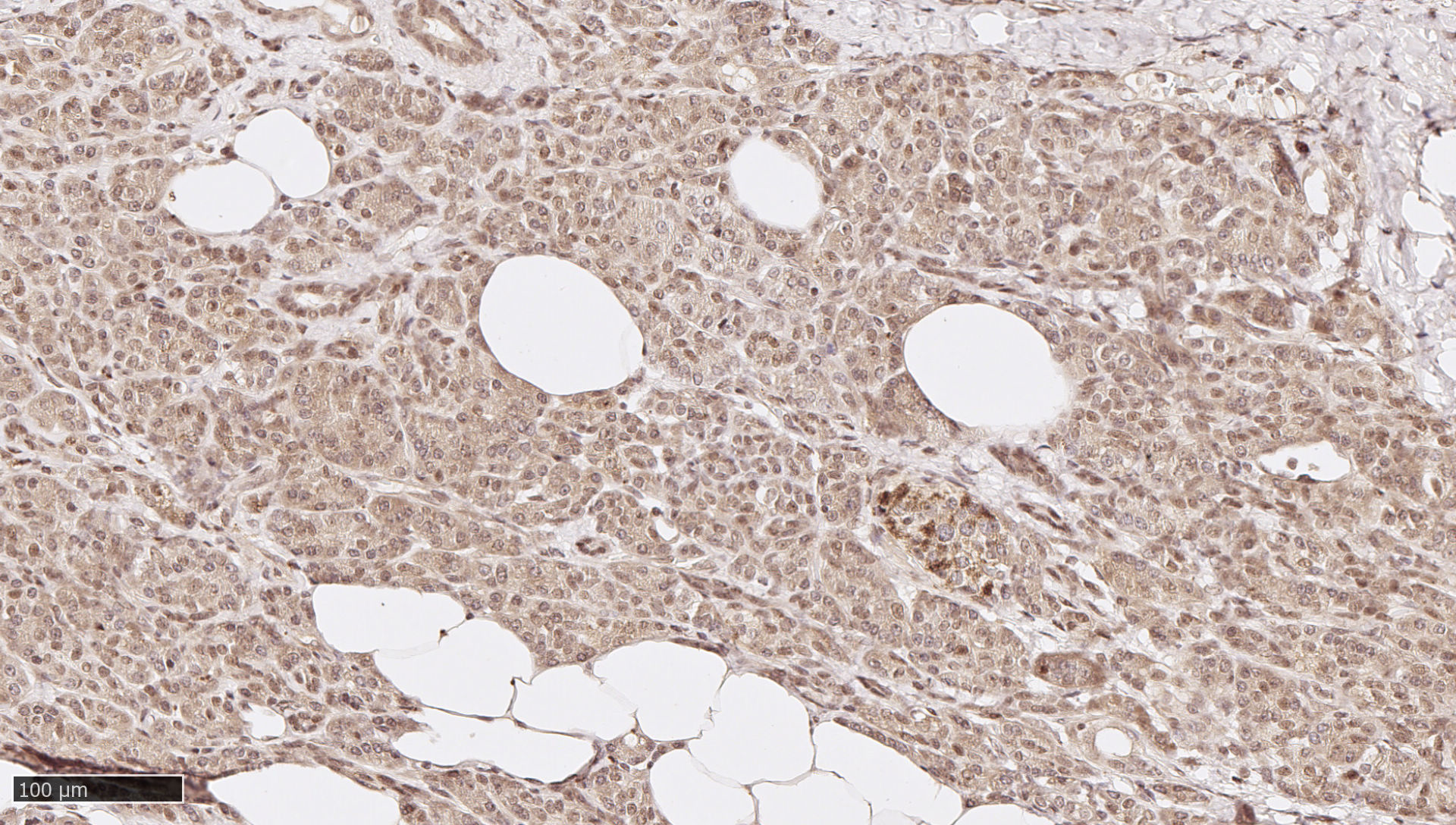

Supplement: S2 Fig — Hes1- Pancreatic Cancer. (TIF) [file pone.0240973.s002.tif]

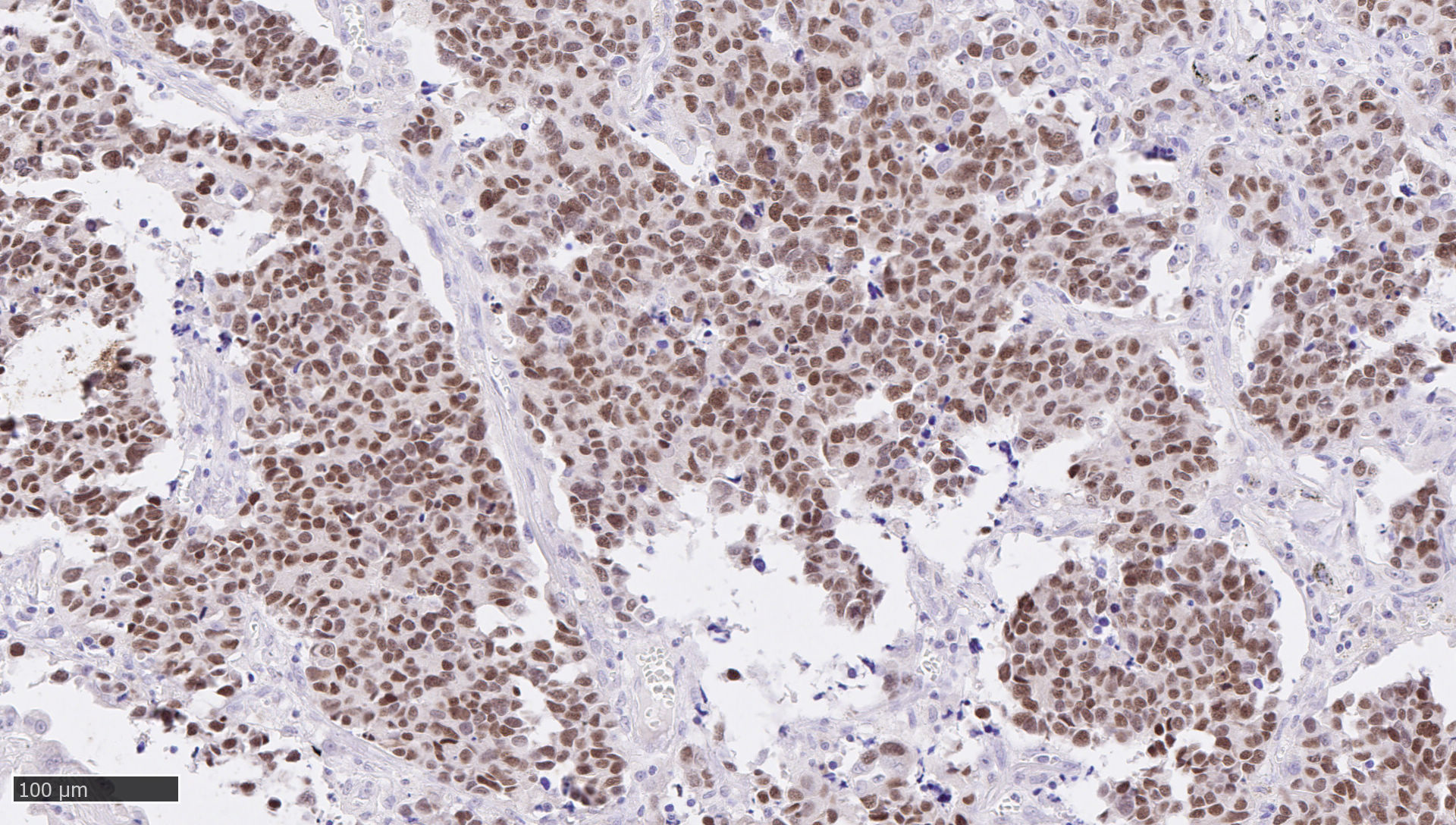

Supplement: S3 Fig — Ascl1- Small Cell Lung Cancer. (TIF) [file pone.0240973.s003.tif]

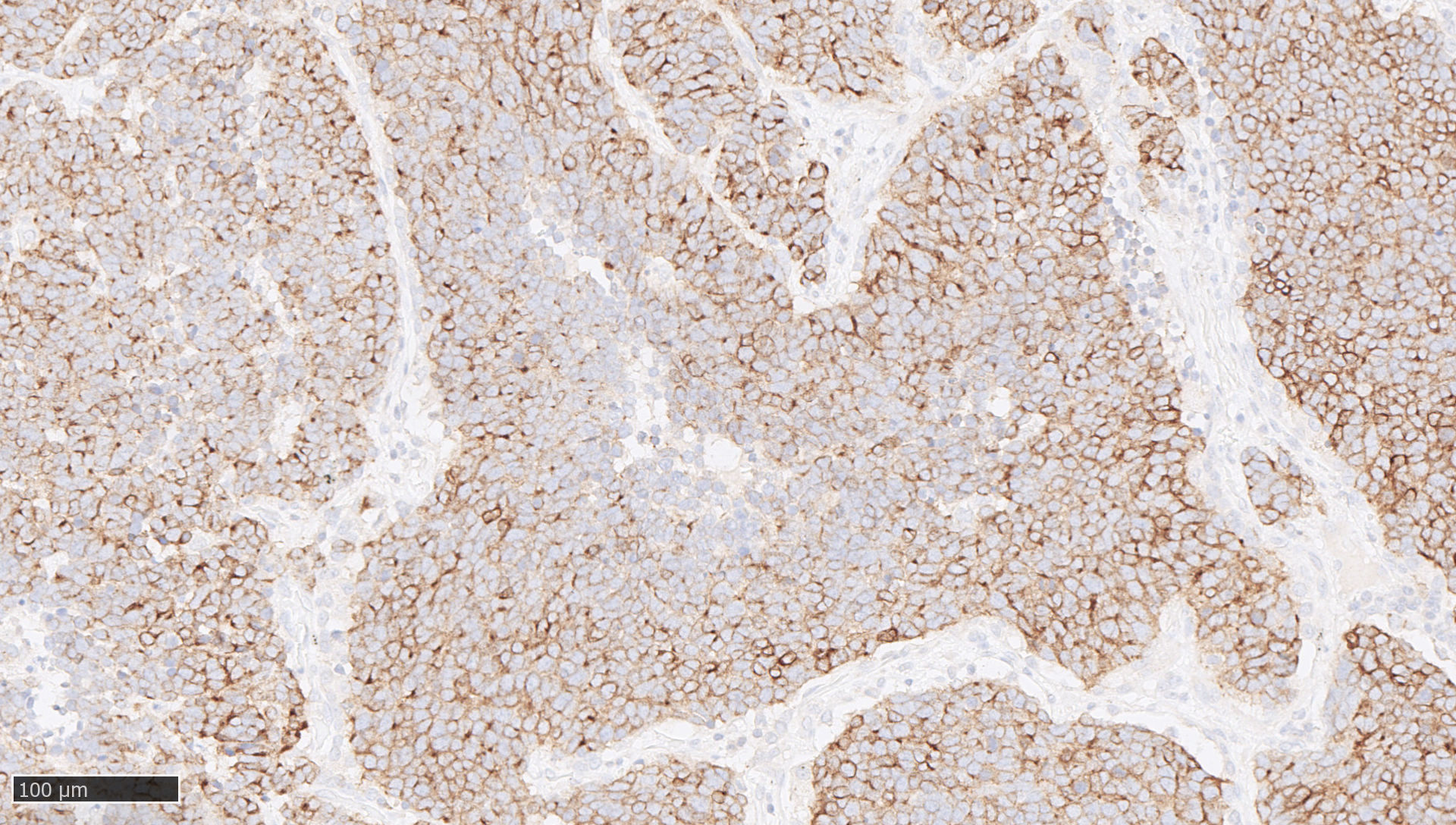

Supplement: S4 Fig — DLL3- Small Cell Lung Cancer. (TIF) [file pone.0240973.s004.tif]

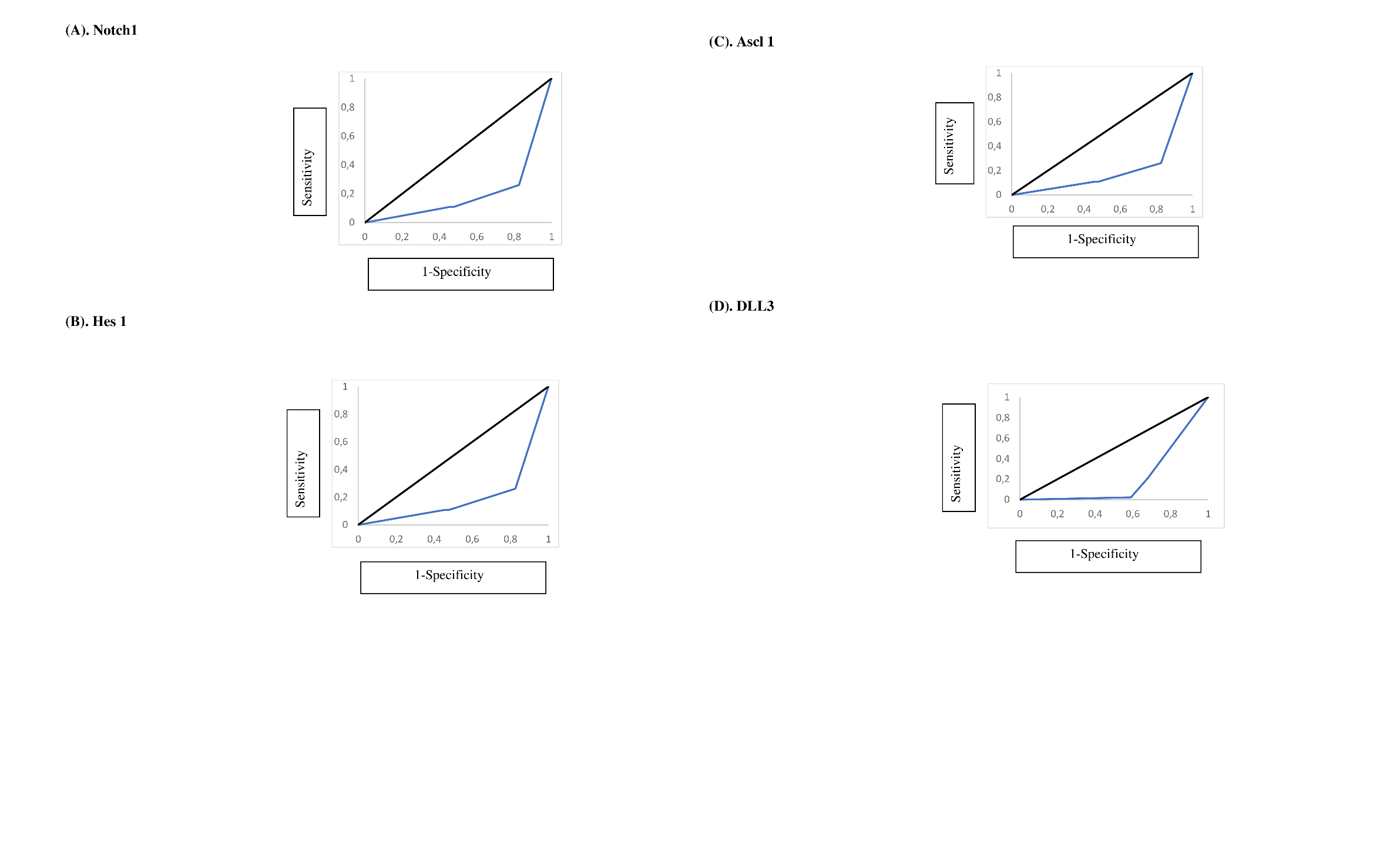

Supplement: S5 Fig — The receiver operating characteristic (ROC) curve analysis for each biomarker Notch 1 (A), Hes1 (B), Ascl1 (C), DLL3 (D), with sensitivity to platinum-doublet chemotherapy as the outcome of interest. (TIF) [file pone.0240973.s005.tif]

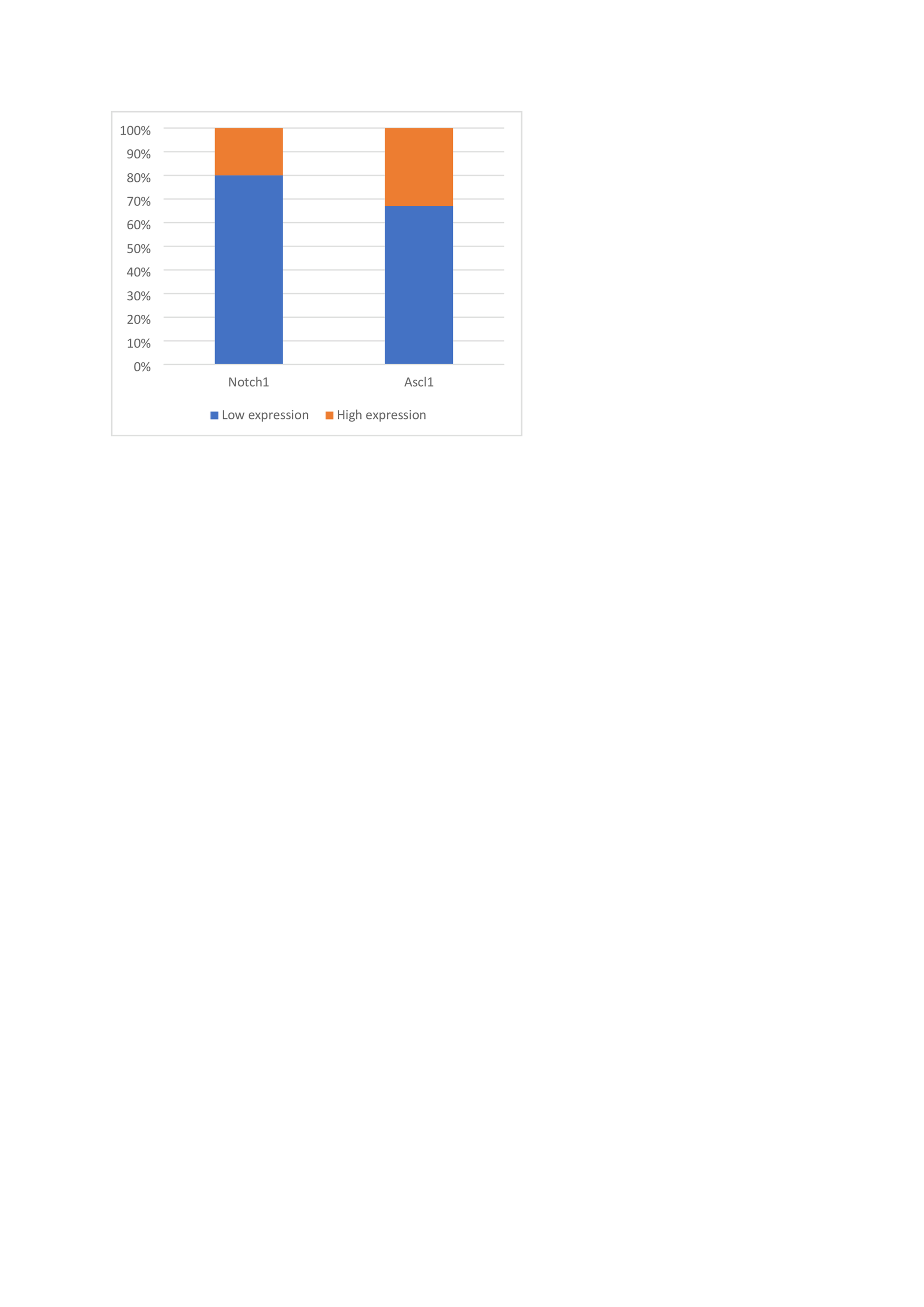

Supplement: S6 Fig — (TIF) [file pone.0240973.s006.tif]
